# On the complexity of Minimum Path Cover with Subpath Constraints for multi-assembly

**DOI:** 10.1186/1471-2105-15-S9-S5

**Published:** 2014-09-10

**Authors:** Romeo Rizzi, Alexandru I Tomescu, Veli Mäkinen

**Affiliations:** 1 Department of Computer Science, University of Verona, Italy; 2 Helsinki Institute for Information Technology HIIT, Department of Computer Science, University of Helsinki, Helsinki, Finland

**Keywords:** multi-assembly, RNA-Seq, minimum path cover, directed acyclic graph, network flow, min-cost circulation

## Abstract

**Background:**

Multi-assembly problems have gathered much attention in the last years, as Next-Generation Sequencing technologies have started being applied to mixed settings, such as reads from the transcriptome (RNA-Seq), or from viral quasi-species. One classical model that has resurfaced in many multi-assembly methods (e.g. in Cufflinks, ShoRAH, BRANCH, CLASS) is the Minimum Path Cover (MPC) Problem, which asks for the minimum number of directed paths that cover all the nodes of a directed acyclic graph. The MPC Problem is highly popular because the acyclicity of the graph ensures its polynomial-time solvability.

**Results:**

In this paper, we consider two generalizations of it dealing with integrating constraints arising from long reads or paired-end reads; these extensions have also been considered by two recent methods, but not fully solved. More specifically, we study the two problems where also a set of subpaths, or pairs of subpaths, of the graph have to be entirely covered by some path in the MPC. We show that in the case of long reads (subpaths), the generalized problem can be solved in polynomial-time by a reduction to the classical MPC Problem. We also consider the weighted case, and show that it can be solved in polynomial-time by a reduction to a min-cost circulation problem. As a side result, we also improve the time complexity of the classical minimum weight MPC Problem. In the case of paired-end reads (pairs of subpaths), the generalized problem becomes NP-hard, but we show that it is fixed-parameter tractable (FPT) in the total number of constraints. This computational dichotomy between long reads and paired-end reads is also a general insight into multi-assembly problems.

## Introduction

### Background

The last years have witnessed Next-Generation Sequencing technologies applied to mixed settings in which the input sample consists of different, but highly related, genomic sequences. A major problem in this setting is to assemble the NGS reads produced from these different sequences, problem called *multi-assembly *[1].

An emblematic example is the multi-assembly of the expressed transcripts of a gene from RNA-Seq reads [2,3]. The RNA transcripts of a gene are concatenations of exons, which can be shared among them, and whose length is typically much longer than the short read length. The RNA-Seq technology has proved essential in characterizing gene regulation and function, understanding development, disease, and disorders, including cancer [4-7]. The most popular tool for multi-assembly of RNA-Seq reads is Cufflinks [8], but the great interest in the community has led to a recent proliferation of methods and tools, such as [9-20]. Another example is the multi-assembly of NGS reads from viral quasi-species [21]. Since many viruses, such as HIV or HCV, encode their genomes in RNA rather than DNA, they lack DNA polymerase and are unable to repair mistakes in their genomes as they reproduce. Over the course of infection, the mistakes made in the replication of the virus are passed down to descendants, producing a family of related variants of the original viral genome, referred to as quasi-species. Among all of the new quasi-species produced, some may be more virulent than others, and it is of great epidemiological interest to identify them. Methods for this problem include [22-27].

The vast majority of the multi-assembly tools are *genome-guided*, in the sense that they have access to one reference genome. Consequently, the analysis proceeds by aligning the reads to this reference, and constructing one of two major graph models. In the first, called an *overlap graph*, the nodes stand for reads and the edges stand for overlaps between reads. This model is employed both for RNA-Seq reads (by Cufflinks [8]), and for pyrosequencing reads from a viral population (ShoRAH [25,26]). In the second model, called a *splicing graph *and used mainly for RNA-Seq reads, the nodes stand for contiguous stretches of DNA present entirely in some transcript (*pseudo-exons*); its edges stand for reads spanning two pseudo-exons and indicate that they are consecutive in some transcript. This model is employed by most of the other methods for the multi-assembly of RNA-Seq reads [9-20]. Since both graph models arise from alignments to a reference sequence, they are also directed and acyclic (DAGs). Moreover, the nodes and the edges of the graph are weighted according to the observed coverage, and different strategies exist for integrating them into the formulation of the multi-assembly problem. For example, in Cufflinks [8], the weight of an edge reflects the belief that its two endpoints originate from *different *transcripts, and is computed using the percent-spliced-in metric proposed in [28].

### Motivation

Given an overlap or a splicing DAG, many methods [8,19,20,25-27] model the multi-assembly problem as a Minimum Path Cover Problem; these include the well-known tool for RNA-Seq reads Cufflinks [8]. A *path cover *in a directed graph *G *is a set of paths which cover all the nodes of *G*. A *minimum path cover *(MPC) is a path cover of minimum cardinality. Often, the edges of the DAG are weighted, and one is then interested in a minimum weight MPC. Even though this problem is in general NP-complete (a path cover has cardinality 1 if and only if the directed graph has a Hamiltonian path), it is solvable in polynomial time on DAGs [29]. This fact is one of the main reasons why the MPC Problem has attracted so much interest. Therefore, it makes sense to extend it with other biological information, while maintaining its polynomial-time solvability.

In this paper we consider additional information arising from paired-end reads or long reads. Observe that, currently, both graph models and the associated MPC Problem include constraints only on *pairs *of nodes which must be *consecutive *in the (same) genomic sequence. However, on the one hand, most sequencers produce paired-end reads; these two reads correspond to nodes that must be in the same genomic sequence, but they are no longer consecutive in it. On the other hand, Third-Generation Sequencing technologies, like Pacific Biosciences [30], produce long reads whose length is in the range of thousands of base-pairs. If properly error-corrected, they introduce additional constraints on the *sequences *of nodes which must appear as consecutive in the same assembled genomic sequences. In the case of a splicing graph, such additional constraints can be introduced even from short reads completely overlapping a short middle pseudo-exon (such as in the case of alternative donor/acceptor sites [31]).

Two different problem formulations have been recently proposed to better guide the multi-assembly using paired-end or long reads. In the first [20], a partial assembly of the RNA transcripts is assumed (*transfrags*), and the following problem, which we call *Minimum Path Cover with Subpath Constraints (MPC-SC)*, is proposed. Given a DAG *G *and a set of subpaths in *G *(the transfrags, or the long reads), we are asked to find a MPC such that each given subpath is contained completely in some path of the path cover. In [20], the authors consider in fact the weighted version of the problem, and propose a polynomial-time reduction to the classical weighted MPC Problem. However, their reduction is incomplete as it does not deal with the case when two subpaths *P*_1 _and *P*_2 _are such that a suffix of *P*_1 _is a prefix of *P*_2_. In the second formulation [19], given a DAG *G *and a set of paired-end RNA-Seq read alignments to the nodes of *G*, we are asked to find a minimum path cover whose paths contain all given paired-end reads. We call this problem *Minimum Path Cover with Paired Subpaths Constraints (MPC-PSC)*. In [19], the authors tackle the MPC-PSC Problem by modeling it as the NP-complete set cover problem.

## Results and discussion

In this paper, we solve both the MPC-SC and the MPC-PSC Problem. Namely, we state the MPC-SC Problem more generally than in [20], and give a correct and robust polynomial-time reduction of it to the classical MPC Problem on a DAG. Denote by *n *the number of nodes of the input DAG, by *m *its number of edges, by *c *the total number of subpath constraints, and by *N *the sum of their lengths. Constructing this reduction to the classical MPC Problem requires a pre-processing step, which, if implemented trivially, takes *O*(*c*^2^*n*^2^) time; however, we can reduce that to *O*(*N *+ *c*^2^) by use of a suffix tree construction suitable for large alphabets [32], and of an optimal-time algorithm for computing all pairs longest suffix-prefix overlaps [33,34]. The complexity of solving Problem MPC-SC thus becomes $O\left(N+c^{2}+\sqrt{\left(n+c\right)}\left(n^{2}+c\right)\right)$.

We also consider the weighted version of Problem MPC-SC, and show that it can be solved in time *O*(*N *+ (*n *+ *c*)^2 ^log(*n *+ *c*) + (*n *+ *c*)(*m *+ *c*)) by a reduction to a min-cost circulation problem on a network with flow lower bounds only [35]. Moreover, we prove that the MPC-PSC Problem itself is NP-complete, but we show that it is fixed-parameter tractable (FPT) in the total number of constraints on the DAG.

As a side result of this paper, we obtain a simple algorithm for the classical minimum weight MPC Problem running in time *O*(*n*^2^log *n *+ *nm*), based on a recent reduction to a network flow problem [36]. This improves the current best bound *O*(*n*^2 ^log *n *+ *nt*(*G*)), where $t\left(G\right)\in \left\{m,m+1,\ldots ,\left(\begin{array}{c} n\\ 2 \end{array}\right)\right\}$ is the number of edges in the transitive closure of *G*, arising from the reduction in [29].

In view of this computational dichotomy between paired-end reads and long reads/transfrags, an alternative title of this paper could have been "Long reads are better than paired-end reads in multi-asssembly problems". In fact, in the experiments we conducted for our own tool for RNA-Seq multi-assembly Traph [37,38], we fed Cufflinks [8], IsoLasso [10], SLIDE [12] and Traph both with single-end and paired-end reads, but did not notice any significant change in the multi-assembly accuracy. Nevertheless, an immediate solution to the negative result concerning the complexity of the MPC-PSC Problem could be to simply transform paired-end reads into long reads by a local assembly method which fills the gap between them, such as [39,40].

As a preliminary experiment, in the Supplementary Material we show the solutions of Problem MPC-SC on simulated RNA-Seq data from six cancer-related genes. These results are compared to the ground truth, and to Cufflinks' solutions (given that Cufflinks uses the classical MPC model). These preliminary results indicate that, thanks to the additional long read constraints to the MPC problem, Problem MPC-SC reports more transcripts than Cufflinks, and they are generally more accurate.

Both MPC-SC and MPC-PSC Problems are natural extensions of the classical MPC problem, and can be applied to any graph model for multi-assembly, such as an overlap graph or a splicing graph. The MCP Problem has received great interest in the multi-assembly community, and pair-end reads, long reads, or transfrags are either already, or expected to be easily available in the near future. Our positive result concerning the MPC-SC Problem, and the two proposed solutions for the MPC-PSC Problem, give efficient ways to incorporate additional information that an NGS pipeline can provide. Moreover, all of our solutions are based on easy to implement reductions, and resort to well-known problems in combinatorial optimization, for which there are many existing solvers.

Independently and parallel to this work, [41] gave analogs of our Thm. 4 and Lemma 2 for Problem MPC-PSC.

## Methods

### A faster algorithm for the weighted Minimum Path Cover (MPC) Problem

Given a directed graph *G*, we say that a family $P=\left\{P_{1},\ldots ,P_{k}\right\}$ of paths in *G *is a *path cover *of *G *if every *v ∈ V *(*G*) belongs to some *P_i_*. Throughout this paper, we let *n *stand for the number of vertices of *G *and *m *stand for the number of edges of *G*. A *minimum path cover *(MPC) of *G *is a path cover of *G *of minimum cardinality. If each edge *e *of *G *has a non-negative weight *w*(*e*), then a *minimum weight minimum path cover *is a minimum path cover  P which minimizes the sum of the weights of the edges of the paths of  P, that is, $\Sigma_{P\in P}$ Σ*_e∈P_w*(*e*).

**Figure 1:**
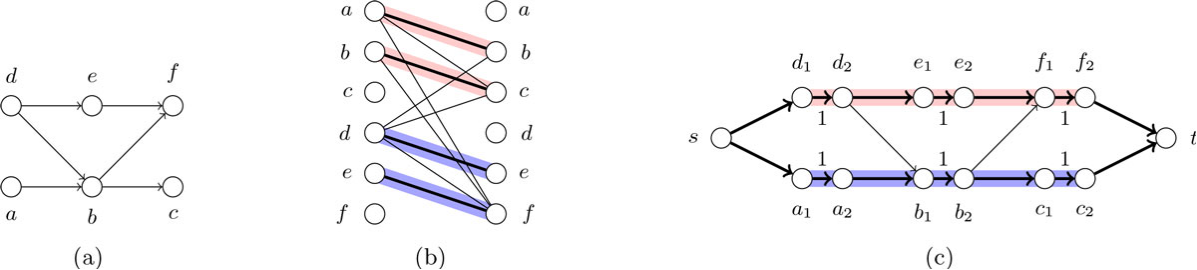
**In Fig**. 1(a), an input DAG *G*. In Fig. 1(b), the reduction to the maximum matching problem: a bipartite graph *B*(*G*) having as vertices two copies of *V*(*G*) and an edge between the first copy of *v*_1 _*∈ V*(*G*) and the second copy of *v*_2 _*∈ V*(*G*) iff there is a directed path in *G *from *v*_1 _to *v*_2_. The edges of a maximum matching of *B*(*G*) are highlighted, and a MPC for *G *is obtained by putting *v*_1 _and *v*_2 _in the same path there is an edge between *v*_1 _and *v*_2 _and is selected by the maximum matching. In Fig. 1(c), a network flow *N*(*G*) corresponding to *G*; the labels '1' on some edges are the lower bounds on that edges; all other edges have lower bound 0. The min-flow on *N*(*G*) has value 2; the edges with flow value 1 are highlighted; any decomposition of this flow into paths gives a MPC.

A well-known result on path covers in directed acyclic graphs (DAGs) is Dilworth's theorem [42], which equates the minimum number of paths in a path cover to the maximum cardinality of an anti-chain (this cardinality is sometimes called *width*); an anti-chain is a set of nodes with no directed path between any two of them. A constructive proof of this theorem, due to Fulkerson [29], shows that the MPC problem can be reduced to a maximum matching problem in a bipartite graph, as follows. Given a directed graph *G*, let *T*(*G*) denote the transitive closure of *G*, that is, the digraph obtained from *G *by repeatedly adding, until no longer possible, an edge (*u, v*) whenever (*u, v*) *∉ E*(*G*) but there exist *w ∈ V *(*G*) such that (*u, w*), (*w, v*) *ϵ E*(*G*); we let *t*(*G*) denote the number of edges of *T*(*G*). Note that if *G *is a DAG, then *T*(*G*) can be computed in time *O*(*t*(*G*)). Fulkerson showed that a MPC can be obtained by computing a maximum matching in a bipartite graph associated to *G*, having two copies of *V *(*G*) as nodes and the edges of *T*(*G*) as edges (see Figures 1(a) and 1(b)). Therefore, using the Hopcroft-Karp maximum matching algorithm, a MPC can be computed in time $O\left(\sqrt{n}t\left(G\right)\right)$[43]. To compute a minimum weight MPC, the same bipartite graph can be constructed, having edge weights induced from path weights in *G*. A minimum weight MPC corresponds to a minimum weight maximum matching on this graph, which can be computed in time *O*(*n*^2^ log *n *+ *nt*(*G*)) [44].

A recent solution for the MPC Problem reduces it instead to a min-flow problem [36], as follows. Each node of *G *is replaced by an arc with lower bound 1 (all other edges of *G *have lower bound 0), and a new global source *s *and sink *t *are added to *G *and connected to all sources and sinks of *G*, respectively (see Figures 1(a) and 1(c)). A min-flow on this digraph is a flow of minimum value satisfying all lower bounds. The value of the min-flow on this network equals the maximum size of an anti-chain of *G*, and any decomposition of it into paths gives a MPC [36]. A decomposition of a flow on a DAG into paths can be computed in time linear in the number of edges, by traversing the edges used by the flow [45]. A min-flow problem can be solved by two applications of a max-flow algorithm [45]. Therefore, using the recent result on max-flows [46], this approach finds a MPC in time *O*(*nm*).

If in the unweighted case, the complexity of the method of [36] is incomparable with the complexity of solving the MPC Problem by a maximum matching problem, in the weighted case, the method of [36] leads to one of improved complexity. This is obtained by an algorithm for the following restricted variant of the min-cost circulation problem [45,47]: given a directed graph, and a flow lower bound for each edge and a cost per flow unit for each edge, the task is to find a circulation of minimum total cost satisfying all lower bounds. A *circulation *is a function assigning a flow value to each edge such that the flow conservation property is satisfied for all nodes; consequently, the flow network cannot have sources or sinks.

To solve the minimum weight MPC Problem, we extend the reduction in [36] by associating to the edges either cost 0, if they correspond to the nodes of *G *or are incident to *s *or *t*; or their weight in *G*, if they correspond to edges of *G*. Moreover, we add a new edge from *t *to *s *with lower bound 0 and having as cost the sum of all edge weights (plus a positive constant if all are 0). This implies that all min-cost circulations induce a min-flow (removing the edge from *t *to *s*), and thus, by [36], induce also a MPC that is of minimum weight; obviously, vice versa, a minimum weight MPC induces a min-cost circulation on the constructed flow network.

There are many algorithms and solvers for the min-cost circulation problem, with various time complexity upper bounds [47], for example *O*(*nm *log log *C *log(*nK*)) [48], where *C *is the maximum edge bound, and *K *is the maximum cost. If edges have only lower bounds, as in our case, the min-cost circulation problem can be solved in time *O*(*n *log *C*(*m *+ *n *log *n*)) [35]; since we have *C *= 1, this reduces to *O*(*n*^2 ^log *n *+ *nm*). Therefore, we have the following theorem.

**Theorem 1 ***A minimum weight MPC of a DAG with n nodes and m edges can be computed in time O*(*n*^2^log *n + nm*), *by a reduction to a min-cost circulation problem*.

### The new problem formulations

We first consider the problem arising from long reads, or from transfrags. We introduce a slight generalization of a path cover of a DAG *G*, namely a set of paths which cover only a given subset $V^{\prime }$ of the nodes. We are also given a subset $E^{\prime }$ of the edges of *G*, and a family of subpaths $P^{in}$ in *G *that all have to be entirely covered by some path of the path cover. We could have modeled each edge constraint in $E^{\prime }$ as a path of length 1 in $P^{in}$, but for clarity, we keep these separate. Formally, we have:

### Minimum Path Cover with Subpath Constraints (MPC-SC) Problem INPUT: **A DAG *G *and**

1 A subset $V^{\prime }$ of *V *(*G*)

2 A subset $E^{\prime }$ of *E*(*G*)

3 A family $P^{in}=\left\{P_{1}^{in},\ldots ,P_{t}^{in}\right\}$ of directed paths in *G*

### **TASK: **Find a minimum number *k *of directed paths $P_{1}^{sol},\ldots ,P_{k}^{sol}$ in *G *such that

1 Every node in $V^{\prime }$ occurs in some $P_{{}^{i}}^{sol}$

2 Every edge in $E^{\prime }$ occurs in some $P_{{}^{i}}^{sol}$

3 Every path *P^in ^ϵ $P^{in}$* is entirely contained in some $P_{i}^{sol}$

We call the elements of the sets $V^{\prime }$,$E^{\prime }$, $P^{in}$*constraints*, and we say that the *k *paths in a solution *satisfy *these constraints.

**Figure 2:**
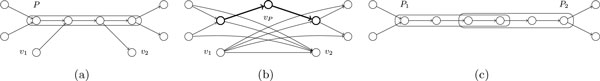
**In Fig**. 2(a), subpath constraint *P *, in Fig. 2(b) the reduction of [20] which replaces path *P *by node *v_P _*connected to the end points of *P *, removes all internal nodes of *P *, and adds all transitive edges from and to *v*_1 _and *v*_2_. In Fig. 2(c), a case not covered by the reduction in [20].

Let us briefly argue that the solution in [20, Sec. 2.4.1. and 2.4.2] for MPC-SC Problem (without the generalization at points 1 and 2) is not complete. (Actually, [20] tackles the Minimum Weight MPC with Subpath Constraints Problem--see below--, but the weights are not relevant for this discussion.) The idea of [20] is to reduce this problem to the classical MPC problem. Consequently, each subpath constraint *P *is modeled by a single edge having the same endpoints as *P *, which is subdivided by introducing a node *v_P _*in the middle (which must be covered by the MPC). The connections between the first or last node of *P *and the other nodes of the DAG are maintained, but since the internal nodes of *P *can no longer be required to be covered by the path cover, they are removed. Moreover, for all nodes *v*_1 _and *v*_2 _such that there is a path between *v*_1 _and *v*_2 _in the DAG using a proper subpath of *P *, a new transitive edge (*v*_1_, *v*_2_) is added. However, this reduction is missing the case in which two subpath constraints *P*_1 _and *P*_2 _are such that a suffix of *P*_1 _is a prefix of *P*_2_. As a matter of fact, our proof will show that the most problematic case is when also a suffix of different length of *P*_1 _is a prefix of some other subpath constraint *P*_3 _(see Figure 2 and the proof of Lemma 1).

In the second problem, we consider the weighted case, with one further generalization, as follows. As also noted by [20], in practice the paths in the path cover should start only in source nodes or in a specific subset of other nodes of *G*; similarly for their ending nodes. For example, in our method for the multi-assembly of RNA-transcripts [37,38], these nodes are identified when there is a sharp increase/decrease in read coverage in the middle of an exon, indicating the start/end of a transcript.

### Minimum Weight Minimum Path Cover with Subpath Constraints (MW-MPC-SC) Problem

**INPUT: **A DAG *G *and

1 A subset $V^{\prime }$ of *V *(*G*)

2 A subset $E^{\prime }$ of *E*(*G*)

3 A family $P^{in}=\left\{P_{1}^{in},\ldots ,P_{t}^{in}\right\}$ of directed paths in *G*

4 A superset *S *of the sources of *G*, and a superset *T *of the sinks of *G*

5 A weight *w*(*e*) for each *e ∈ E*(*G*)

**TASK: **Find a minimum number *k *of directed paths $P_{1}^{sol},\ldots ,P_{k}^{sol}$ in *G *such that

1 Every node in $V^{\prime }$ occurs in some $P_{i}^{sol}$

2 Every edge in $E^{\prime }$ occurs in some $P_{i}^{sol}$

3 Every path *P ^in ^∈ *$P^{in}$ is entirely contained in some $P_{i}^{sol}$

4 Every path $P_{i}^{sol}$ starts in a node of *S *and ends in a node of *T*

5 $\underset{i\in \left\{1,\ldots ,k\right\}}{\sum }\underset{\text{edge}e\in P_{i}^{sol}}{\sum }w\left(e\right)$ is minimum among all tuples of *k *paths satisfying properties 1-4

When only paired-end reads are available, each such pair of reads corresponds to a pair of subpaths that must both be covered by the same path in the path cover. Formally, we have:

### Minimum Path Cover with Paired Subpath Constraints (MPC-PSC) Problem

**INPUT: **A DAG *G *and

1 A subset $V^{\prime }$ of *V *(*G*)

2 A subset $E^{\prime }$ of *E*(*G*)

3 A family $P^{in}=\left\{\left(P_{1,1}^{in},P_{1,2}^{in}\right),\ldots ,\left(P_{t,1}^{in},P_{t,2}^{in}\right)\right\}$ of pairs of directed paths in *G*

**TASK: **Find a minimum number *k *of directed paths $P_{1}^{sol},\ldots ,P_{k}^{sol}$ in *G *such that

1 Every node in $V^{\prime }$ occurs in some $P_{i}^{sol}$

2 Every edge in $E^{\prime }$ occurs in some $P_{i}^{sol}$

3 For every pair $\left(P_{j,1}^{in},P_{j,2}^{in}\right)\in P^{in}$, there exists such $P_{i}^{sol}$ that both $P_{j,1}^{in}$ and $P_{j,2}^{in}$ are entirely contained in $P_{i}^{sol}$

### The MPC with Subpath Constraints (MPC-SC) Problem

#### The unweighted case

In this section, we reduce the MPC-SC Problem to the classical MPC Problem. We describe our reduction as a sequence of commented algorithmic steps.

**Step 1**. for every $\left(u,v\right)\in E^{\prime }$ do: $V^{\prime }:=V^{\prime }\backslash \left\{u,v\right\}$;

If the MPC has a path *P *covering the arc (*u, v*), then *P *also covers both *u *and *v*. Therefore, the constraints *u, v *can be dropped from $V^{\prime }$ (if present).

**Step 2**. for every path $P_{j}^{in}\in P^{in}$ and for every edge $\left(u,v\right)\in P_{j}^{in}$ do:

$$V^{\prime }:=V^{\prime }\backslash \left\{u,v\right\};E^{\prime }:=E^{\prime }\backslash \left\{u,v\right\};$$

Similarly to Step 1, if the MPC has a path *P *covering a subpath $P_{j}^{in}\in P^{in}$ , then *P *also covers every node and edge of $P_{j}^{in}$, thus these constraints can be dropped from $V^{\prime }$ and $E^{\prime }$ (if present).

**Step 3**. while there exist two paths $P_{i}^{in}$ and $P_{j}^{in}$ in $P^{in}$ such that $P_{i}^{in}$ is contained in $P_{j}^{in}$ do:

$$P^{in}:=P^{in}\backslash P_{i}^{in};$$

After this step, no subpath constraint is completely included into another; this is key for the correctness of Step 4 below.

**Step 4**. while there exist two paths $P_{i}^{in}$, $P_{j}^{in}\in P^{in}$ such that a suffix of $P_{i}^{in}$ is a prefix of $P_{j}^{in}$ do:

let $P_{i}^{in}$, $P_{j}^{in}\in P^{in}$ be as above and with the common part (i.e., the suffix of $P_{i}^{in}$ which is a prefix of $P_{j}^{in}$) the *longest *possible;

let $P_{new}^{in}$:= the path $P_{i}^{in}\cup P_{j}^{in}$ which starts as $P_{i}^{in}$ and ends as $P_{j}^{in}$;

$$P^{in}:=\left(P^{in}\backslash \left\{P_{i}^{in},P_{j}^{in}\right\}\cup \left\{P_{new}^{in}\right\}\right);$$

In this step, we merge paths sharing a suffix/prefix. We do this iteratively, at each step merging that pair of paths for which the shared suffix/prefix is longest possible. The correctness of this step is guaranteed by Lemma 1 below.

**Lemma 1 ***If the MPC-SC Problem on an instance *$\left(G,V^{\prime },E^{\prime },P^{in}\right)$*admits a solution with k paths, then also the problem instance transformed by applying Steps 1-4 admits a solution with k paths, and this solution also satisfies the original constraints *$V^{\prime }$, $E^{\prime }$, $P^{in}$.

*Proof *The correctness of Steps 1-3 was argued next to their introduction. Assume that $G,V^{\prime },E^{\prime },P^{in}$ have been transformed by these first three steps, and let $P_{i}^{in},P_{j}^{in}\in P^{in}$ be such that their common part (i.e., the suffix of $P_{i}^{in}$ which is a prefix of $P_{j}^{in}$) is *longest *possible. Suppose that the original problem admits a solution $P^{sol}=\left\{P_{1}^{sol},\ldots ,P_{k}^{sol}\right\}$ such that $P_{i}^{in},P_{j}^{in}$ are covered by different solution paths say $P_{a}^{sol}$ and $P_{b}^{sol}$, respectively. We show that the transformed problem admits a solution $P^{*}=\left(\left\{P_{1}^{sol},\ldots ,P_{k}^{sol}\right\}\backslash \left\{P_{a}^{sol},P_{b}^{sol}\right\}\cup \left\{P_{a}^{*},P_{b}^{*}\right\}\right)$, having the same cardinality as P, in which $P_{i}^{in},P_{j}^{in}$ are covered by the same path $P_{a}^{*}$, and $P^{*}$ also satisfies the original constraints $V^{\prime },E^{\prime },P^{in}$.

**Figure 3:**
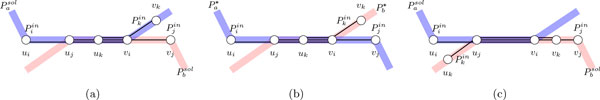
**A visual proof of Lemma 1**.

Suppose that $P_{i}^{in}$ starts with node *u_i _*and ends with node *v_i_*, and that $P_{j}^{in}$ starts with node *u_j _*and ends with node *v_j _*. Let (cf. Figures 3(a) and 3(b)):

• $P_{a}^{*}$ be the path obtained as the concatenation of the path $P_{a}^{sol}$ taken from its starting node until *v_i _*with the path $P_{b}^{sol}$ taken from *v_i _*until its end node (so that $P_{a}^{*}$ covers both $P_{i}^{in}$ and $P_{j}^{in}$).

• $P_{b}^{*}$ be the path obtained as the concatenation of the path $P_{b}^{sol}$ taken from its starting node until *v_i _*with the path $P_{a}^{sol}$ taken from *v_i _*until its end node.

We have to show that the path cover $P^{*}=\left(\left\{P_{1}^{sol},\ldots ,P_{k}^{sol}\right\}\backslash \left\{P_{a}^{sol},P_{b}^{sol}\right\}\right)\cup \left\{P_{a}^{*},P_{b}^{*}\right\}$ satisfies the original constraints $V^{\prime },E^{\prime },P^{in}$. Since $P_{a}^{*}$ and $P_{b}^{*}$ use exactly the same edges as $P_{a}^{sol}$ and $P_{b}^{sol}$, then $V^{\prime }$ and $E^{\prime }$ are satisfied. Moreover, the only two problematic cases are when there is a subpath constraint $P_{k}^{in}$ which has *v_i _*as internal node and is satisfied only by $P_{a}^{sol}$, or it is satisfied only by $P_{b}^{sol}$. Denote, analogously, by *u_k _*and *v_k _*the endpoints of $P_{k}^{in}$. From the fact that the input was transformed at Step 3, $P_{i}^{in}$ and $P_{j}^{in}$ are not completely included in $P_{k}^{in}$.

**Case 1. **$P_{k}^{in}$ is satisfied only by $P_{a}^{sol}$ (Figures 3(a) and 3(b)). Since $P_{i}^{in}$ is not completely included in $P_{k}^{in}$, *u_k _*is an internal node of $P_{i}^{in}$; thus, a suffix of $P_{i}^{in}$ is prefix also of $P_{k}^{in}$. From the fact that the common part between $P_{i}^{in}$ and $P_{j}^{in}$ is longest possible, we have that vertices *u_j _, u_k _, v_i _*appear in this order in $P_{i}^{in}$. Thus, $P_{k}^{in}$ is also satisfied by $P_{b}^{*}$, since *u_k _*appears after *u_j _*on *P^i^*.

**Case 2. **$P_{k}^{in}$ is satisfied only by $P_{b}^{sol}$, and it is not satisfied by $P_{a}^{*}$ (Figure 3(c)). This means that $P_{k}^{in}$ starts on $P_{b}^{sol}$ before *u_j _*and, since it contains *v_i_*, it ends on $P_{b}^{sol}$ after *v_i_*. From the fact that $P_{j}^{in}$ in not completely included in $P_{k}^{in}$, *v_k _*is an internal node of $P_{j}^{in}$, and thus a suffix of $P_{k}^{in}$ equals a prefix of $P_{j}^{in}$. This common part is now longer than the common suffix/prefix between $P_{i}^{in}$ and $P_{j}^{in}$, which contradicts maximality of the suffix/prefix between $P_{i}^{in}$ and $P_{j}^{in}$. This proves the lemma.

The remaining steps can be seen as analogous to the reduction in [20].

**Step 5**. for every path $P_{i}^{in}\in P^{in}$ do:

say $P_{i}^{in}$ starts in node *s *and ends in node *t*;

$$P^{in}:=P^{in}\backslash \left\{P_{i}^{in}\right\};$$

$$E\left(G\right):=E\left(G\right)\cup \left\{\left(s,t\right)\right\};$$

$$E^{\prime }=E^{\prime }\cup \left\{\left(s,t\right)\right\};$$

In this step, we represent each subpath constraint by an edge constraint. Its correctness is guaranteed by the fact that by now, no two subpath constraints are such that a suffix of the first is a prefix of the second. We should stress out that if there are more paths with the same endpoints, we may add parallel edges to the DAG. However, in Step 6 below these parallel edges will be transformed into parallel paths of length 2, rendering the DAG simple again.

**Step 6**. for every edge $e\in E^{\prime }$ do:

$$E^{\prime }:=E^{\prime }\left\{e\right\};$$

subdivide the edge *e *by introducing a node *v_e _*in the middle of it;

$$V^{\prime }:=V^{\prime }\cup \left\{e\right\};$$

At this point, we have transformed all subpath constraints into edge constraints. The edge constraints can be modeled as node constraints by simply subdividing each edge and introducing a new node in the middle of it; this node is then added to $V^{\prime }$.

**Step 7**. *G*:= *T*(*G*)

We replace *G *by its transitive closure, since in Step 8 below we are going to remove from *G *all vertices not in $V^{\prime }$.

**Step 8**. Remove from *G *all nodes not in $V^{\prime }$;

Since only the nodes in $V^{\prime }$ have to be covered by the paths in the path cover, we remove all other nodes. This is correct, since, at Step 7 above, we introduced all edges between nodes *v *and $v^{\prime }$ such that $v^{\prime }$ was reachable from *v *through some nodes not in $V^{\prime }$.

**Step 9**. Compute a MPC for the resulting graph *G*;

This can be done by any method discussed previously.

**Step 10**. Postprocess the paths obtained at Step 9 above by reverting the transformations executed at Steps 1-8, in reverse order.

**Theorem 2 ***Problem MPC-SC on a graph with n nodes, m edges, c subpath or edge constraints, and with N being the sum of subpath constraint lengths, can be solved by solving the classical MPC Problem in a graph with O*(*n + c*) *nodes and O*(*n*^2 ^+ *c*) *edges. This graph can be computed in time O*(*N + c*^2 ^+ n^2^), *thus the complexity of Problem MPC-SC is *$O\left(N+c^{2}+\sqrt{\left(n+c\right)}\left(n^{2}+c\right)\right)$.

*Proof *The complexity of the pre-processing phase is dominated by Steps 3 and 4. Step 3 can be solved by first building a (generalized) suffix tree on the concatenation of subpath constraints with a distinct symbol #*_i _*added after each constraint sequence $P_{i}^{in}$. This can be done in *O*(*N*) time even on our alphabet of size *O*(*n*) [32].

Then one can do as follows during depth-first traversal of the tree: If a leaf corresponding to the suffix starting at the beginning of subpath constraint $P_{i}^{in}$ has an incoming edge labeled by only #*_i _*and its parent has still other children, then the constraint is a substring of another constraint and must be removed (together with the leaf).

For Step 4, we compute all pairs longest suffix-prefix overlaps between the subpath constraints using an *O*(*N *+ *c*^2^) time algorithm in [33, Theorem 7.10.1, page 137], [45] with [32] as a subroutine for the sake of large alphabet. The output can be casted to a double-linked list *L *containing elements of the form (*i, j*, len, prev*_i_*, next*_i_*, prev*_j _*, next*_j _*) in decreasing order of the overlap length, len, between constraints $P_{i}^{in}$ and $P_{j}^{in}$. Pointers prev*_i_*, next*_i_*, prev*_j_*, and next*_j_* tell the previous/next occurrences of the tuple having *i *as the first element and *j *as the second element, respectively. Then popping the first tuple from *L *tells us the first constraints to merge, and following prev*_∗ _*and next*_∗ _*pointers we can remove all overlaps no longer relevant for next mergings; when removing, we need to make sure the nested double-linked lists formed by the prev*_∗ _*and next*_∗ _*pointers are also updated. Continuing like this until the list *L *is empty gives all the overlaps in total *O*(*c*^2^) time. Notice that the new merged constraints do not need to be separately taken into account in overlap computation; no completely new overlaps can be created due to Step 3.

Merging itself requires a similar linked list structure being a special case of unionfind: All the constraints are represented as double-linked lists with node numbers as elements. Merging can be done by linking the double-linked lists together, removing the extra overlapping part from the latter list and redirecting its start pointer to point inside the newly formed merged list. When finished with merging, the new constraints are exactly those old constraints whose start pointers still point to the beginning of a node list. The complexity of merging is thus *O*(*N*).

#### The weighted case

To solve the MW-MPC-SC Problem, we build on the reduction in [36] to a network flow problem. This reduction will allow the addition of edge weights and of constraints on the starting/ending nodes of the solution paths. Note that these constraints *S *and *T *cannot be included in the reduction of the MPC Problem to a bipartite matching problem. Moreover, the heuristic in [20, Sec. 2.4.2] of arbitrarily extending the paths in a minimum weight MPC towards sources/sinks cannot be proved to be correct.

**Figure 4:**
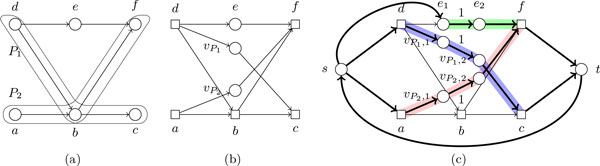
**In Fig. 4(a), an input DAG *G *with two subpath constraints *P*_1 _and *P*_2_; we take**. $V^{\prime }=V\left(G\right)$, $E^{\prime }=0̸$, *S *= {*a, d, e*} and *T *= {*f, c*}; weights are not drawn. In Fig. 4(b), the graph transformed by Steps 1-6; the vertices still in $V^{\prime }$ are drawn as circles, other vertices as squares. In Fig. 4(c), the reduction to a min-cost circulation problem; the edges with flow lower bound 1 are labeled as '1'; other edges have flow lower bound 0. In a min-cost circulation of value 3, all highlighted edges have flow value 1, except for (*f, t*) with flow value 2, and (*t, s*) with value 3. Any decomposition of the min-cost circulation into 3 paths gives the solution for Problem MW-MPC-SC.

Given an input $\left(G,V^{\prime },E^{\prime },S,T,w\right)$ for the MW-MPC-SC Problem, we pre-process the graph *G *by Steps 1-6 of the unweighted case (shown in the previous section). After this pre-processing, we have correctly modeled all subpath constraints by node constraints. On the transformed graph *G*, we then do a similar reduction as for Thm. 1 (see Figure 4):

1 We replace each node $v\in V^{\prime }$ by an edge (*v*_1_, *v*_2_) such that all in-neighbors of *v *are now in-neighbors of *v*_1_, and all out-neighbors of *v *are now out-neighbors of *v*_2_. If node *v *was introduced at Step 6 to model an edge coming from a subpath constraint *P *, then the cost per unit of flow of (*v*_1_, *v*_2_) is the sum of the weights of the edges of *P *; otherwise, it is 0.

2. For each edge *e *of *G*, if *e *is an original edge of *G*, we set its flow lower bound to 0 and its cost per unit of flow to *w*(*e*); otherwise we set both to 0.

3. The global source *s *has out-going edges precisely to the nodes in the set *S*, and the global sink *t *has in-coming edges precisely from the nodes in *T *; we also add the edge (*t, s*). All edges incident to *s *or *t *have flow flower bound 0 and cost 0, except for the edge (*t, s*) having as cost the sum of all edge weights (plus a positive constant if all are 0). This guarantees, like before, that any min-cost circulation is also a min-flow.

Note that, by reducing to a flow problem, we do not have to perform Steps 7 and 8 anymore, since the coverage constraints are now modeled as flow lower bound constraints. As in the case of Thm. 1, we compute a min-cost circulation on this transformed input *G*, that is, a function *f *: *E*(*G*) *→ *ℕ which satisfies all the flow conservation property for all nodes, satisfies all edge lower bounds, and minimizes $\sum_{e\in E\left(G\right)}f\left(e\right)$. We then decompose the circulation (from which we remove the edge (*t, s*)) into paths, and covert these paths into paths of the original input graph. This is done by reverting the transformations executed at Steps 1-6, in reverse order (as done for the MPC-SC Problem). As before, these paths form a MPC satisfying all constraints, and they also start and end in vertices of *S *and *T *, respectively (because of the way *s *and *t *were connected to the other nodes of the graph). Since these paths arise from a min-cost circulation, then they also form a minimum weight MPC satisfying the input constraints. The flow network has only flow lower bounds, thus we can again apply the algorithm of [49], to get the following:

**Theorem 3 ***Problem MW-MPC-SC on a graph with n nodes, m edges, c subpath or edge constraints, and with N being the sum of subpath constraint lengths, can be solved by reducing it to a min-cost circulation problem on a network with O*(*n + c*) *nodes and O*(*m + c*) *edges, and with flow lower bounds only. This network can be computed in time O*(*N + c*^2 ^+ *m*), *and the complexity of Problem MW-MPC-SC becomes O*(*N* + (*n + c*)^2 ^log(*n + c*) + (*n + c*)(*m + c*)).

### The MPC with Paired Subpaths Constraints (MPC-PSC) Problem

#### The NP-completeness proof

In this section we show that the MPC-PSC Problem is NP-complete. Our reduction is from the NP-complete problem of deciding whether the chromatic number of a graph *G, χ*(*G*), is 3 [50]. We will show that it is actually NP-complete to determine if the MPC-PSC Problem admits a solution with just 3 paths, even on planar DAGs, of width 2, series-parallel, when only paired subpath constraints are imposed, and all subpaths are just edges.

**Figure 5:**
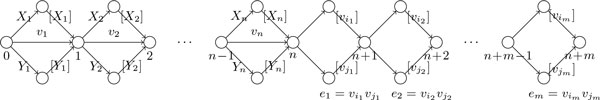
**A reduction from chromatic number 3 to the MPC-PSC Problem**.

Let *G *= (*V, E*) with *V *= {*v*_1_,*..*., *v_n_*} and *E *= {*e*_1_,*..*., *e_m_*} be any non-bipartite graph; our question is whether *χ*(*G*) = 3. We reformulate this question by building up the DAG *P*(*G*) drawn in Figure 5. *P*(*G*) consists of a first stage of *n *blocks corresponding to the *n *vertices of *G*, and a second stage of *m *blocks corresponding to each edge $e_{k}=v_{i_{k}}v_{j_{k}}$ of *G, k ∈ *{1, ..., *m*}. Only some of the nodes and edges of *P *(*G*) have been labeled; when an edge is labeled [*L*], we mean that in the family of paired subpath constraints we have the constraint (*L*, [*L*]).

**Theorem 4 ***Problem MPC-PSC is NP-complete.*

*Proof *We show that the graph *G *= (*V, E*) has *χ*(*G*) = 3 if and only if the DAG *P*(*G*) drawn in Figure 5 admits a solution to Problem MPC-PSC with 3 paths.

 (⇒) Suppose that *χ*(*G*) = 3. Definitely, we need at least three paths to solve *P*(*G*), since the three edges *v*_1_, *X*_1_, *Y*_1 _exiting from node 0 cannot be covered by the same path, and each of them is mentioned in some constraint. By definition, *G *is 3 colorable if and only if *V *can be partitioned into three sets *V_A_, V_B _, V_C _*such that no edge of *G *is contained in any of them. We use these three sets to build up the three solution paths for Problem MPC-PSC as follows: for all *X ∈ *{*A, B, C*}, in the first stage (until node *n*) path *P_X _*picks up all edges labeled with a node in *V_X _*and no edge labeled with a node in *V\V_X _*; next, in the second stage (from node *n *until node *n *+ *m*), *P_X _*picks up those edges $\left[v_{i_{k}}\right]$ such that $v_{i_{k}}$ belongs to *P_X _*. This is possible, since no edge $e_{k}=v_{i_{k}}v_{j_{k}}$ is contained in the same color class, and consequently the two of edges of *P *(*G*) labeled $v_{i_{k}}$ and $v_{j_{k}}$ do not belong to the same path among {*P_A_, P_B _, P_C _*}. Thus, $\left[v_{i_{k}}\right]$ and $\left[v_{j_{k}}\right]$ do not have to be both covered by the same solution path. Therefore, the three paths *P_A_, P_B _, P_C _*satisfy all paired subpath constraints, and are a solution to Problem MPC-PSC.

(⇐) Suppose the DAG *P*(*G*) drawn in Figure 5 admits a solution to Problem MPC-PSC with 3 paths *P_A_, P_B _, P_C _*. Then, we partition *V *into three color classes *A, B, C *by setting *v_i_∈ × *if and only if the edge of *P*(*G*) labeled by *v_i _*(in the first stage from node 0 to node *n*) belongs to *P_X _*, for all *X ∈ *{*A, B, C*}. To see that {*A, B, C*} is indeed a partition of *V *, observe that in each block *k *of the first stage of *P*(*G*), no two paths in {*P_A_, P_B _, P_C _*} can share an edge, since all three edges *v_k _, X_k _, Y_k _*appear in some constraint. Therefore, each edge *v_k _*appears in exactly one of {*P_A_, P_B _, P_C _*}. The proof that the partition {*A, B, C*} is also a proper coloring of *G *encounters no difficulty, as the rationale behind the reduction was illustrated in the forward implication.

**Corollary 1 ***For no ε > 0 there exists a *$\left(\frac{4}{3}-\in \right)$-*approximation algorithm for Problem MPC-PSC unless P=NP. Moreover, the problem is not FPT when parameterized on OPT (the minimum number of paths in a solution)*.

#### The FPT algorithm

In the previous section, we obtained the NP-completeness for the decision problem *OPT *= 3; this rules out a Dynamic Programming approach for Problem MPC-PSC. In this section, we show that if *OPT *= 2, then the problem can be solved in polynomial time. This also leads to an FPT algorithm on the total number of constraints.

For any constraint of the input DAG *G *that is made up of a pair (*P*_1_, *P*_2_) of subpaths of *G*, we may assume that there exists a directed path of *G *completely containing both *P*_1 _and *P*_2_, otherwise, the input instance is infeasible. Given any two constraints *X *and *Y *(*X *and *Y *can be nodes, edges, or pairs of subpaths), we say that *X *and *Y *are *compatible *if there is a directed path of *G *completely containing both *X *and *Y *. We exploit the following structural property:

**Lemma 2 ***Let C be a set of constraints on a DAG G. There exists a directed path P in G which satisfies all constraints in C if and only if any two constraints in C are compatible.*

*Proof *The forward implication is clear from the definition. For the backward implication, recall that the width of a DAG denotes the maximum size of an anti-chain of it. We claim that the union of the constraints in *C *is a DAG of width 1. Indeed, if it were of width 2 it would contain two nodes *v*_1 _and *v*_2 _which are pairwise not reachable by a directed path, thus forming an anti-chain of size 2. Since we assumed that for all pairs (*P*_1_, *P*_2_) of subpaths constraints of *G*, there exists a directed path of *G *completely containing both *P*_1 _and *P*_2_, this implies that *v*_1 _and *v*_2 _belong to two different constraints *X *and *Y *in *C*. Thus, *X *and *Y *are not compatible, a contradiction.

**Theorem 5 ***Given an instance for Problem MPC-PSC, we can decide in polynomial time if OPT = 2, and if so, find the two solution paths. Moreover, Problem MPC-PSC is fixed-parameter tractable (FPT) in the total number C of input constraints.*

*Proof *We build an incompatibility graph from the input constraints: every constraint is represented by a node, and we add an edge between two constraints iff they are *incompatible*. Then, *OPT *= 2 iff this incompatibility graph is bipartite, and the two classes of the bipartition give the two solution paths; this can be done in time *O*(*C*^2^). If *OPT *> 2, then we try all possible ways of partitioning the set of all input constraints (the number of these possibilities is a function only on *C*), and check that each class of the partition consists of pairwise compatible constraints.

## Competing interests

The authors declare that they have no competing interests.

## Authors' contributions

AT conceived the problems and wrote the manuscript. RR, AT and VM contributed to the solutions. All authors read and approved the final manuscript.

## Declarations

Publication of this article was supported by the Academy of Finland under grant 250345 (CoECGR).

This article has been published as part of BMC Bioinformatics Volume 15 Supplement 9, 2014: Proceedings of the Fourth Annual RECOMB Satellite Workshop on Massively Parallel Sequencing (RECOMB-Seq 2014). The full contents of the supplement are available online at http://www.biomedcentral.com/bmcbioinformatics/supplements/15/S9.
